# Total and component forest aboveground biomass inversion via LiDAR-derived features and machine learning algorithms

**DOI:** 10.3389/fpls.2023.1258521

**Published:** 2023-10-26

**Authors:** Jiamin Ma, Wangfei Zhang, Yongjie Ji, Jimao Huang, Guoran Huang, Lu Wang

**Affiliations:** ^1^ College of Forestry, Southwest Forestry University, Kunming, China; ^2^ College of Geography and Ecotourism, Southwest Forestry University, Kunming, China; ^3^ Aisino Xinde Zhitu (Beijing) Technology Co, Beijing, China

**Keywords:** forest total and component AGB, machine learning, LiDAR, validation methods, random forest

## Abstract

Forest aboveground biomass (AGB) and its biomass components are key indicators for assessing forest ecosystem health, productivity, and carbon stocks. Light Detection and Ranging (LiDAR) technology has great advantages in acquiring the vertical structure of forests and the spatial distribution characteristics of vegetation. In this study, the 56 features extracted from airborne LiDAR point cloud data were used to estimate forest total and component AGB. Variable importance–in–projection values calculated through a partial least squares regression algorithm were utilized for LiDAR-derived feature ranking and optimization. Both leave-one-out cross-validation (LOOCV) and cross-validation methods were applied for validation of the estimated results. The results showed that four cumulative height percentiles (*AIH*_30,_ *AIH*_40_, *AIH*_20_, and *AIH*_25_), two height percentiles (*H*_8_ and *H*_6_), and four height-related variables (*H*_mean_, *H*_sqrt_, *H*_mad_, and *H*_curt_) are ranked more frequently in the top 10 sensitive features for total and component forest AGB retrievals. Best performance was acquired by random forest (RF) algorithm, with *R* ^2 = ^0.75, root mean square error (RMSE) = 22.93 Mg/ha, relative RMSE (rRMSE) = 25.30%, and mean absolute error (MAE) = 19.26 Mg/ha validated by the LOOCV method. For cross-validation results, *R* ^2^ is 0.67, RMSE is 24.56 Mg/ha, and rRMSE is 25.67%. The performance of support vector regression (SVR) for total AGB estimation is *R* ^2 = ^0.66, RMSE = 26.75 Mg/ha, rRMSE = 28.62%, and MAE = 22.00 Mg/ha using LOOCV validation and *R* ^2 = ^0.56, RMSE = 30.88 Mg/ha, and rRMSE = 31.41% by cross-validation. For the component AGB estimation, the accuracy from both RF and SVR algorithms was arranged as stem > bark > branch > leaf. The results confirmed the sensitivity of LiDAR-derived features to forest total and component AGBs. They also demonstrated the worse performance of these features for retrieval of leaf component AGB. RF outperformed SVR for both total and component AGB estimation, the validation difference from LOOCV and cross-validation is less than 5% for both total and component AGB estimated results.

## 1 Introduction

The forest is the most important terrestrial ecosystem on Earth, playing a critical role in the global carbon cycle and terrestrial biosphere (He et al., 2007). The aboveground biomass (AGB) of forest is an important parameter that characterizes their carbon sequestration capacity. Because forest biomass affects a range of ecosystem processes, such as carbon and water cycles, energy fluxes, and thus local and regional climate, the development of sustainable forest management strategies requires accurate information on forest AGB (Urbazaev et al., 2018). Quantitative estimates of aboveground forest biomass provide basic data to support the carbon cycle of the global forest biomass system, thus contributing to the development of global carbon reduction policies and climate change mitigation and providing the necessary information for the development of sustainable forest management (Dixon et al., 1994; Cao et al., 2014). Forest AGB is all aboveground living material, and it includes partitioned biomass components like stem, bark, leaves, and branches (Zhang et al., 2011). Biomass components provide important information for forest management decisions. For example, knowledge of crown biomass aids fuel load assessment and strategies of fire management. Although knowledge of the contribution of each component to AGB is crucial for studying forest growth, it is essential to understand how these components interact with each other (Lambert et al., 2005; Saatchi et al., 2007; He et al., 2013). Meanwhile, the saturation problems resulted from the low penetration of short electromagnetic wave in the forest with high AGB level are the bottleneck problems in forest AGB inversion using remote sensing technology, and accurate estimation of biomass components can improve the estimation saturation points in AGB estimation and reduce the uncertainty of carbon sink estimation, which is the key to quantify carbon stock and plays an important role in modern forest and ecosystem management (Jia et al., 2015; Nie et al., 2017). Therefore, accurate forest total AGB and forest AGB components are crucial. However, accurate estimation of forest total AGB and biomass components is still a challenging task in forestry research at present.

Light Detection and Ranging (LiDAR) is a powerful tool for estimating AGB in forests with fine resolution, which provides detailed three-dimensional information about the forest structure by emitting LiDAR pulses that penetrate the canopy. This is closely related to the spatial heterogeneity of forest carbon content and habitat (Asner et al., 2012; Vaglio Laurin et al., 2016). Total and component AGB were investigated and estimated using LiDAR-derived features in previous research (Næsset and Gobakken, 2008; Tsui et al., 2012; He et al., 2013; Cao et al., 2014). A large number of LiDAR-derived features were demonstrated to be useful of predicting biomass; however, they also revealed that the results were site or species dependent (Zhao et al., 2009; Salas et al., 2010). In parametric regression algorithms, the population assumptions of regression models are usually based on linear relationships. However, it is difficult to fully describe the complex non-linear relationship between forest AGB and LiDAR data using traditional statistical regression methods (Zhao et al., 2019; Torre-Tojal et al., 2022). Several studies found that machine learning algorithms, such as random forest (RF) and support vector regression (SVR) that abandoned the population assumptions that did not represent the heterogeneity of forest stands in parametric regression algorithms, performed better than parametric algorithms in forest AGB estimation (Breidenbach et al., 2010; Vauhkonen et al., 2010; Gleason and Im, 2012; Görgens et al., 2015). Therefore, many studies have applied machine learning algorithms for forest AGB estimation. The performance of machine learning algorithms applied in forest AGB estimation using LiDAR-derived features was not fully explored by far, and their capability for accurate AGB estimation has no agreement (Gleason and Im, 2012). Although the machine learning algorithms show a good performance, the large amount of data constrains the direct use features extracted from LiDAR data as input to the inversion models. For forest AGB estimation using remote sensing observations, a key step is to optimize the optimal features from abundant remote sensing observation (Yang et al., 2017). The importance of each extracted feature was interpreted and ranked by Görgens et al. (Görgens et al., 2015); however, the optimization of feature was not fully explored yet; especially, little was known about component AGB estimation.

For machine learning models to estimate forest AGB, the method of testing model performance is important and involves appropriate validation methods to determine the best predictive model. Leave-one-out cross-validation (LOOCV) and cross-validation are popular for validating the results of forest biophysical parameter estimation. However, how effectively can these two validation methods be used and how different are they and what impact do they have on the estimation results? In our knowledge, it is not addressed yet. According to abovementioned research gap, this study focuses on forest total and component AGB retrieval using height-related features extracted from LiDAR by RF and SVR algorithms. Partial least squares regression (PLSR) algorithm combining the advantages of both principal component analysis (PCA) and multiple linear regression (MLR) was used for ranking and selecting the optimal LiDAR features. LOOCV and cross-validation methods were utilized and compared for results validation. By comparing the two validation methods, we aim to improve the reliability of the model assessment, find the most suitable validation strategy for the inversion model and data in the study area, and improve the efficiency of the utilization of computational resources with the expectation that the model performs well in a variety of data scenarios. The objective is to address the effects of the validation methods and also explore the potential of RF and SVR for forest AGB and component biomass inversion and the potential of PLSR algorithm for selecting the optimal LiDAR-extracted features. To be more concise, we use forest total biomass to describe the forest AGB; biomass components to describe the stem, bark, leave, and branch component biomass; and forest AGBs to describe both total and component biomass. It is expected to provide a valuable reference for the selection of the validation methods in machine learning inverse forest biomass and component biomass studies.

## 2 Materials and methods

### 2.1 Study area

**Figure 1:**
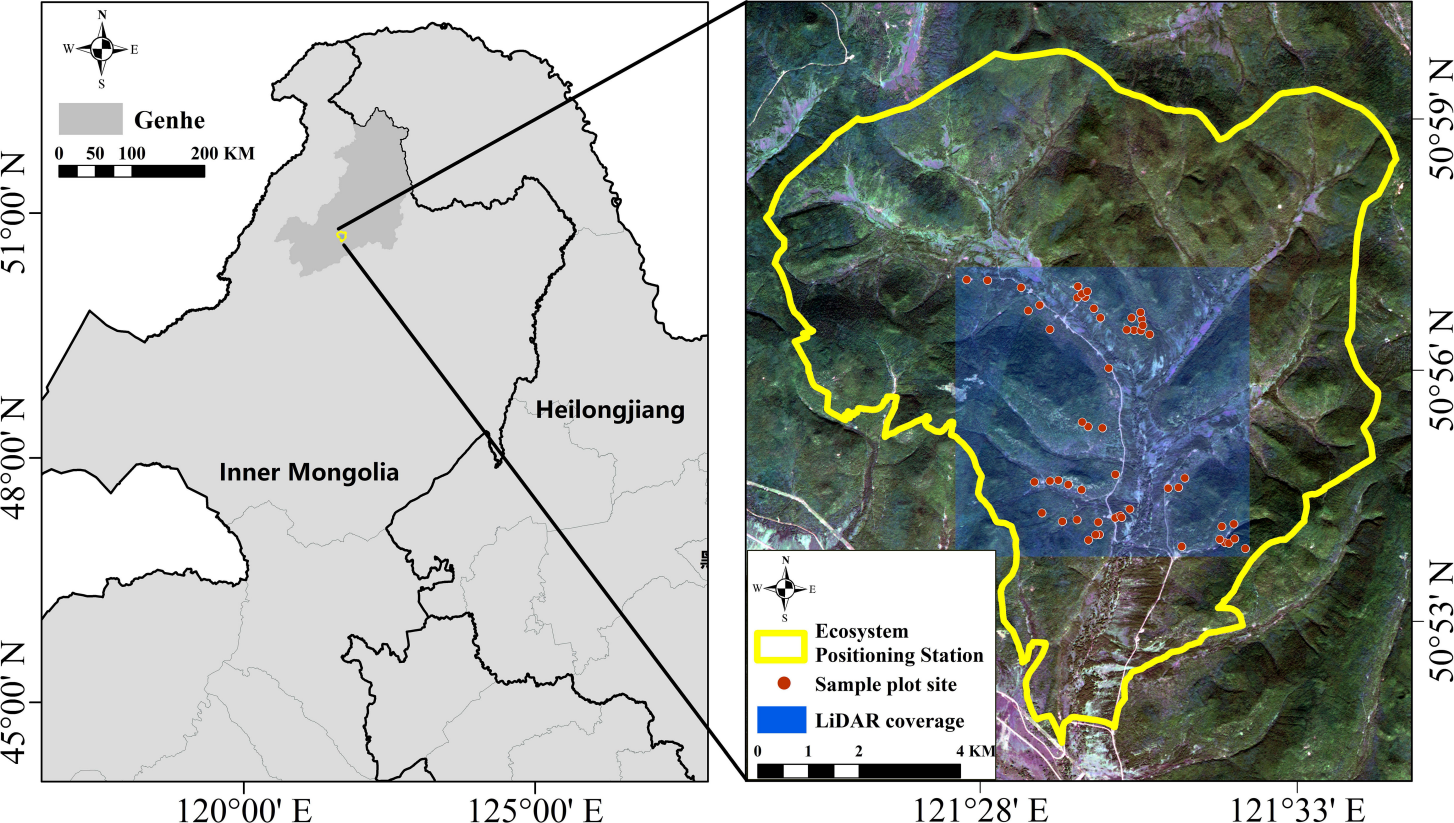
Location and sample plot distribution of study area.

The Daxinganling National Forest Ecosystem Locating Station is the study area, and it is located in Genhe City, Hulunbuir, Inner Mongolia (50°20′N–52°30′N; 120°12′E–122°55′E; **Figure 1**), which is the highest latitude forest ecosystem field scientific observation station in China. Genhe is one of the cities with the highest latitude in China and the lowest average temperature in Inner Mongolia Autonomous Region, with an annual average temperature of −11°C~5°C. The terrain in the study area is comparatively flat, over 80% of the study area with slopes less than 15°. The average elevation is 1,000 m, and the elevation ranges from 700 m to 1300 m. The climate here is a cold-temperate humid forest climate and has some characteristics of continental monsoon climate, a typical area of high latitude permafrost and cold-temperate forest ecosystems. It is cold and wet, with long winters and short summers. The forest cover of the study area is more than 75%, and the main forest type is cold-temperate coniferous forest with complex forest vertical structure. The dominant tree species included in the field-sampled plots are *Larix gmelinii* and *Betula platyphylla*. These tree species covered around 95% of the forest type in the study area. The detailed information regarding tree species coverage is shown in **Supplementary Figure 1**.

### 2.2 Remote sensing data collection and pre-processing

#### 2.2.1 Collection of LiDAR data

In this study, aerial LiDAR data were acquired and utilized to extract features for forest AGB inversion. The LiDAR data were collected during August to September 2012, using the “Yun-5” manned aircraft equipped with a RIEGL LMS-Q680i laser sensor. During this campaign, the average flying altitude of the airborne platform was 2,700 m, with an average flying speed of 220 km/h, and 32 parallel flight tracks were acquired. The laser pulse frequency was 100–200 kHz with a scan angle of ±35° perpendicular to the flight direction. The average point cloud density was 5.6 points/m², and the scan overlap rate was about 80%. The acquired area in this campaign were 213 km². The data format was LAS1.4, and the sensor recorded the three-dimensional coordinate information (x, y, z) of each laser return point, as well as information such as the number of point clouds, intensity, and return type. The detail information of performance of RIEGL LMS-Q680i laser sensor are shown in **Table 1**. The maximum pulse repetition rate was 200 kHz, the maximum scanning frequency was 100 Hz, the wavelength of the pulsed laser was 1,064 nm, and the relative flying height ranged from 200 to 5,000 m. The maximum scanning angle was 75° (**Table 1**).

**Table 1:** The performance of the RIEGL LMS-Q680i laser sensor for LiDAR data collection.

Index	Value
Maximum pulse repetition rate/kHZ	200
Maximum scanning frequency/Hz	100
pulse laser wavelength/nm	1,064
Relative flight height/m	200–5,000
Maximum scanning angle/(°)	75
Point density/(number m^−2^)	>4

#### 2.2.2 Preprocessing of LiDAR data

**Figure 2:**
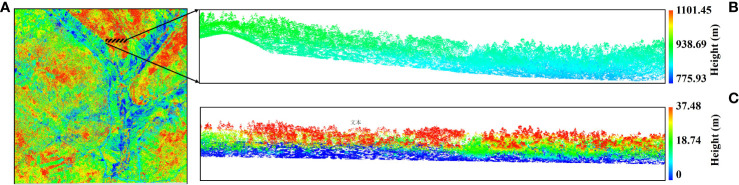
Point cloud data preprocessed results. **(A)** Raw point cloud data of the study area. **(B)** Point cloud data before normalization. **(C)** Point cloud data after normalization.

The raw LiDAR data were processed by the research group of Chinese Academy of Forestry (CAF) through three main steps, namely, full waveform decomposition, geocoding, and boresight calibration (Pang et al., 2016). The preprocessing steps for the LiDAR data in this research include point cloud denoising, point cloud filtering, point cloud classification, and normalization of LiDAR point cloud data. Here, the normalization removes the influence of terrain undulations on the elevation values of the point cloud data, requiring that the range of the DEM has an intersection area with the range of the point cloud data, and the process is to subtract the corresponding DEM elevation value found from the elevation value Z for each point. LiDAR360 software was used for the left preprocessing of LiDAR data, and, then, the preprocessed LiDAR data were used to extract features for forest AGB estimation (**Figure 2**). The results of the pre-processed LiDAR point cloud data were shown as **Figure 2**.

#### 2.2.3 LiDAR feature extraction

After data preprocessing, a total of 56 LiDAR feature parameters were extracted (**Supplementary Table 1**). The extraction of these parameters was based on previous research findings (Liu et al., 2018; Michałowska and Rapiń, 2021; Zhou et al., 2022). To determine the density of point cloud data based on height, the data were split into 10 equally sized height layers for points above 2 m. Within each layer, the ratio of the number of points to the total number of points was calculated, resulting in a density feature parameter. This method offers a way to analyze point density at different heights, which can help reveal patterns and features of the object being scanned.

### 2.3 Field campaign and processing of the collected plot measurements

#### 2.3.1 Collection of plot data

The ground plot data used in this study were collected through field surveys conducted in the Genhe during August in 2012 and 2013 and used for training and validation of the forest AGB inversion models. The collected plots included 25 fixed plots of 40 m × 40 m surveyed in 2012 and 18 plots of 45 m × 45 m investigated in 2013. Differential Global Positioning System (GPS) was used to locate the four corner coordinates of each plot, and the errors of plot boundary and position were controlled within 1 m. In each sample plot, diameter at breast height (DBH), tree height (H), and tree species were recorded for trees with DBH ≥ 5 cm.

#### 2.3.2 Aboveground biomass calculation

Forest total biomass and component biomass of each tree were calculated by allometric equations and component equations published by the State Forestry Administration of China (State Forestry Administration of China, 2016a; State Forestry Administration of China, 2016b). Then, the total AGB and component AGBs of each plot were calculated by the sum of each tree in the plot with normalization by area of each sample plot (Li et al., 2015).

**Figure 3:**
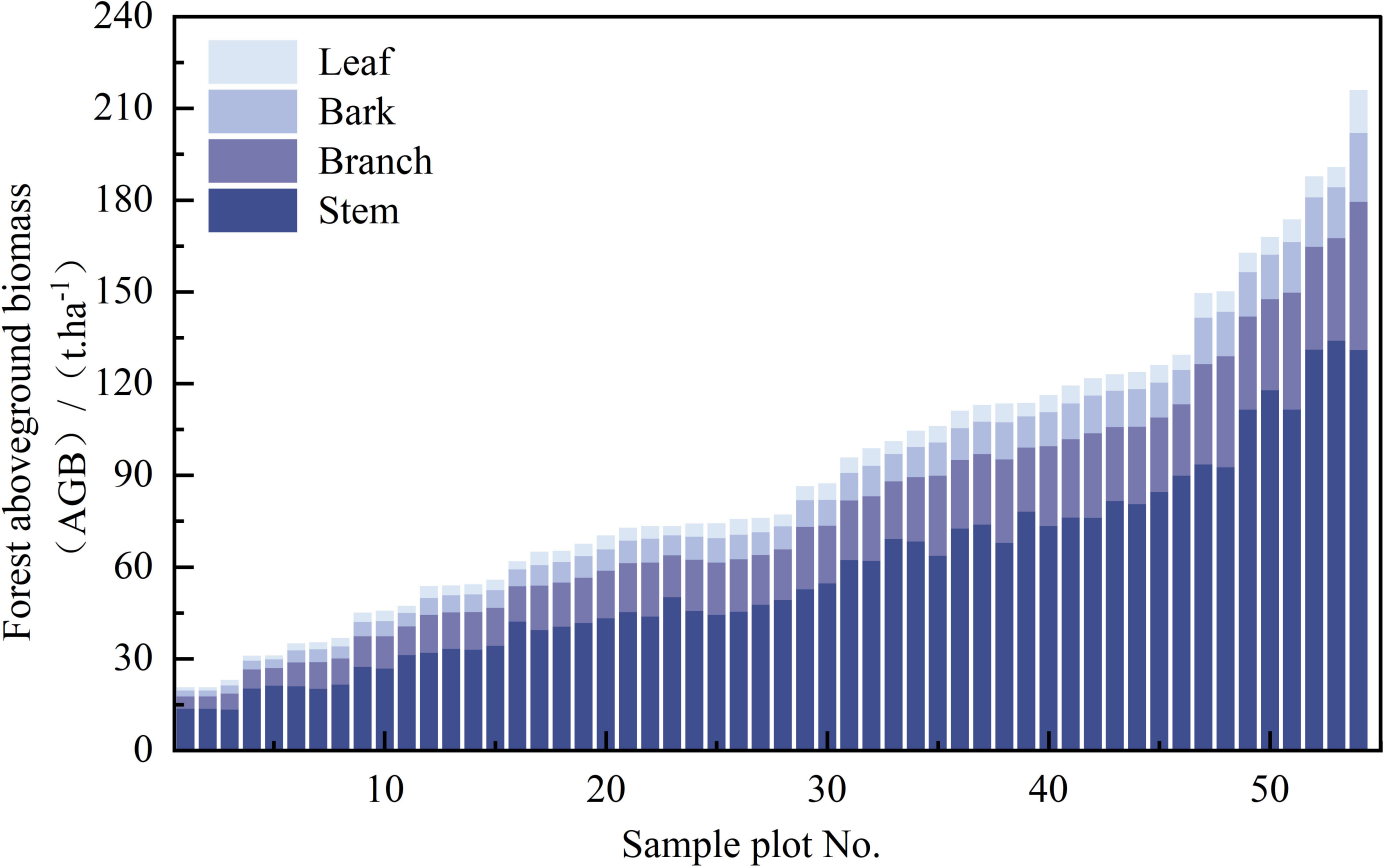
The distribution of total and component AGB for each plot.

In this study, two tree species, namely, *Larix gmelinii* and *Betula platyphylla*, were involved, and the corresponding equations for AGB calculation are shown in **Table 2**. **Figure 3** and **Table 3** show the statistics of calculated forest total and component AGBs.

**Table 2:** Equations for calculating total and component AGBs.

AGB	Equations	*R* ^2^
Total AGB ( $M_{A}$ )	$M_{Larch}=060848D^{2.01549}H^{0.59146}(DBH\geq 5.0cm)$ $M_{Birch}=0.06807D^{0.10850}H^{0.52019}(DBH\geq 5.0cm)$	*Larix gmelinii*: 0.9690 *Betula platyphylla*: 0.9550
Stem biomass ( $M_{Stem}$ )	$M_{Stem}=\frac{1}{{ɡ}_{1}+{ɡ}_{2}+{ɡ}_{3}}\times M_{A}$	*Larix gmelinii*: 0.9701 *Betula platyphylla*: 0.9545
Bark biomass ( $M_{Bark}$ )	$M_{Bark}=\frac{{ɡ}_{1}}{{ɡ}_{1}+{ɡ}_{2}+{ɡ}_{3}}\times M_{A}$	*Larix gmelinii*: 0.8817 *Betula platyphylla*: 0.8678
Branch biomass ( $M_{Branch}$ )	$M_{Branch}=\frac{{ɡ}_{2}}{{ɡ}_{1}+{ɡ}_{2}+{ɡ}_{3}}\times M_{A}$	*Larix gmelinii*: 0.8513 *Betula platyphylla*: 0.9545
Leaf biomass ( $M_{Leaf}$ )	$M_{Leaf}=\frac{{ɡ}_{3}}{{ɡ}_{1}+{ɡ}_{2}+{ɡ}_{3}}\times M_{A}$	*Larix gmelinii*: 0.7439 *Betula platyphylla*: 0.6311

$M_{Larch}$ is the AGB of a Larch (kg); $M_{Birch}$ is the AGB of a Birch (kg); $M_{A}$ is the estimated AGB of tree species (kg); H is the height of the tree (m); D is the diameter of a tree measured at breast height (cm); $M_{Stem}$ , $M_{Bark}$ , $M_{Branch}$ , and $M_{Leaf}$ are the AGB of stem, bark, branches, and leaves of each tree, respectively; $g_{1}$ , $g_{2}$ , and $g_{3}$ are the proportional functions of bark, branches, and leaves, respectively, relative to stem biomass of 1.

Larix gmelinii: ${ɡ}_{1}=0.36742DBH^{-0.16892}H^{-0.17313}$ , ${ɡ}_{2}=2.30634DBH^{0.72188}H^{-1.45081}$ , and ${ɡ}_{3}=1.57804DBH^{0.19527}H^{-1.36274}$ .

*Betula platyphylla*: ${ɡ}_{1}=0.53498DBH^{0.09004}H^{-0.46520}$ , ${ɡ}_{2}=1.05167DBH^{0.66925}H^{-1.04662}$ , and ${ɡ}_{3}=0.61793DBH^{0.17097}H^{-0.88182}$

**Table 3:** Statistics of calculated total and component AGBs.

Type	Mean/(t ha^−1^)	Maximum/(t ha^−1^)	Minimum/(t ha^−1^)	Standard deviation/(t ha^−1^)	Standard error/(t ha^−1^)
Total AGB	92.80	203.53	20.40	46.32	7.06
Stem biomass	61.14	142.80	10.41	32.66	4.98
Branch biomass	18.12	35.72	5.77	8.51	1.30
Bark biomass	9.15	17.77	2.37	4.27	0.65
Leaf biomass	4.39	8.30	1.26	1.63	0.25

### 2.4 Methodology

**Figure 4:**
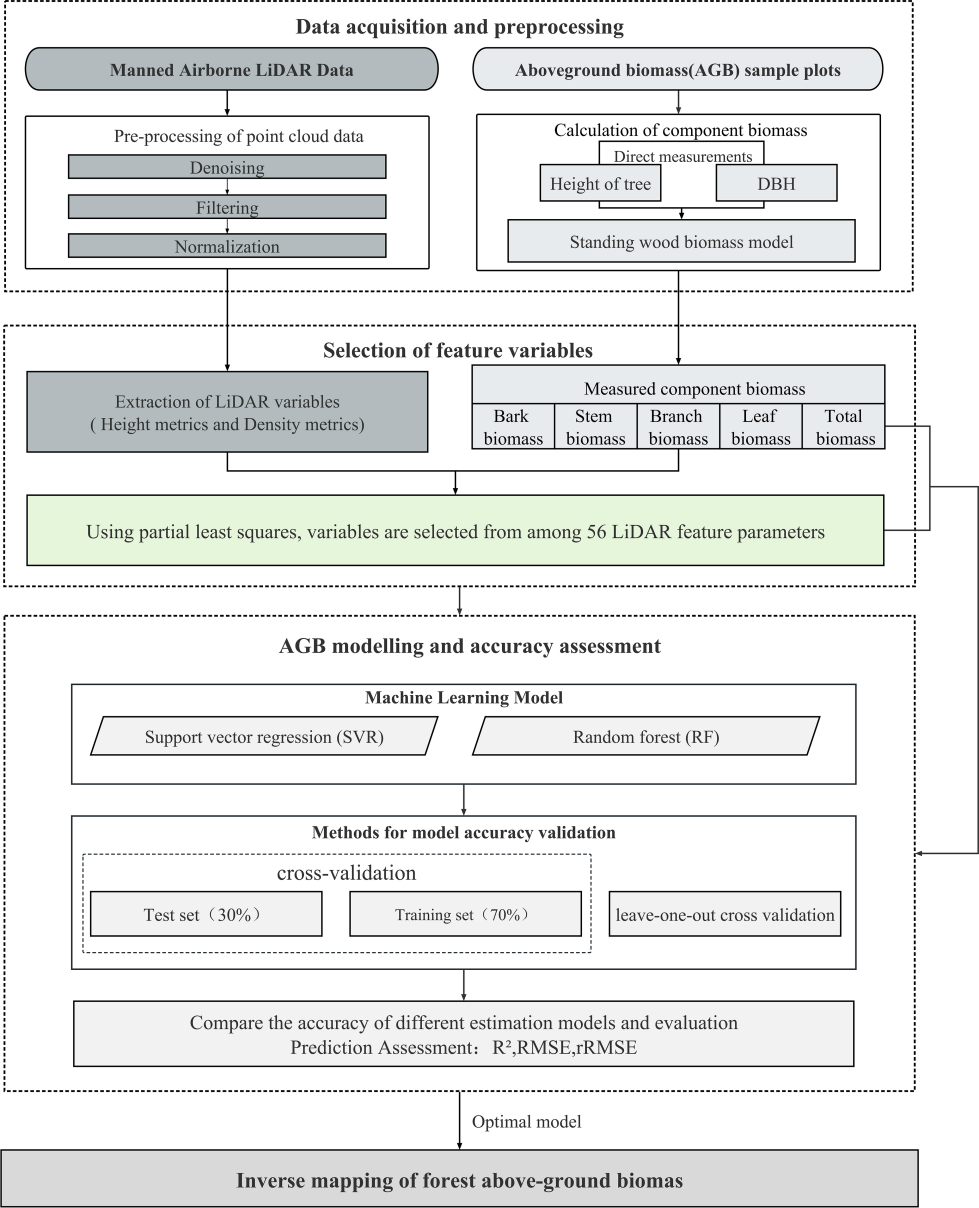
Strategy of identifying suitable LiDAR-derived features (PLSR) and suitable forest total and component AGBs modeling approach (RF and SVR).

**Figure 4** illustrates the framework of this study; first, LiDAR-derived features were extracted as independent variables, and the total and component AGB of each plot were calculated worked as dependent variables; second, PLSR algorithm was used for optimal independent variables selection; third, SVR and RF algorithms were trained for estimating forest total and component AGB; finally, validation and comparative analysis were performed (**Figure 4**).

#### 2.4.1 LiDAR-derived feature selection using partial least squares regression

PLSR was a multivariate statistical analysis algorithm. It could achieve quantitative analysis in the case of multiple correlation of independent variables and could easily exclude the noise in the independent variables. It combined the advantages of PCA and MLR and had outstanding advantages in solving the problems that were difficult to analyze in MLR and in dealing with the problem of multiple cointegration among independent variables. PLSR algorithm optimizes linear regression models to project the input LiDAR-derived features and output AGB into new orthogonal spaces with better predictive capabilities, and it was effective for large number of explanatory LiDAR-derived features that were often not independent of each other. For the procedure of selecting the variables, in this study, by maximizing the covariance between projected LiDAR-derived features and the total forest biomass or component biomass, the orthogonal loading matrix could be solved, and, then, the number of explanatory features for AGB or component biomass was significantly reduced. During the procedure, VIP [variable importance in projection; Equation (1)] values were calculated to optimize the LiDAR-derived measurements. The higher the VIP value, the more significant the independent variable was to the dependent variable; if all independent factors had identical explanatory power over dependent variable, then all VIP values were 1.


(1) $$VIP=\sqrt{\frac{k}{\sum_{h=1}^{n}r^{2}\left(y,c_{h}\right)}\sum_{h=1}^{n}r^{2}\left(y,c_{h}\right)w_{hj}^{2}}$$

where k is the number of independent variables, $c_{h}\;$ is the principal component extracted from the independent variable of interest, $r\left(y,c_{h}\right)$ is the correlation coefficient between the dependent variable and the principal component, denoting the explanatory power of the principal component for y , and $w_{hj}$ is the weight of the independent variable on the principal component.

#### 2.4.2 Machine learning approaches for forest AGB inversion

RF and SVR were selected and employed to predict forest total AGB and component AGB in this study. RF was based on decision trees, and the original training samples were randomly sampled with put-back by bootstrap algorithm, and the samples that are not included in the decision trees were used as test samples. For the regression problem, the predicted values of each decision tree in the RF were used to average the final predicted values (Breiman, 2001). In this study, we used the sklearn package of Python software to train and validate RF model for predicting forest total biomass and biomass components. The maximum feature variable (mtry) and the number of decision trees (ntree) were set as 100 and 15 during the model training procedure.

SVR seeks to obtain the best promotion ability based on a small number of samples by finding the optimal balance between the model’s complexity and learning capacity (Drucker et al., 1997); SVR includes non-linear regression and linear regression algorithms; the basic idea of non-linear regression algorithm is to introduce a suitable kernel function in the sample dataset to map the data from low-dimensional space to high-dimensional space; then, the non-linear problem in low-dimensional space is converted into a linear problem in high-dimensional space. The linear regression is performed in this high-dimensional space, aiming to find the best fit line of the data, i.e., the hyperplane with the highest number of points that can accurately predict the data (Chen et al., 2010; Xue et al., 2010; Xiong and Li, 2019). In this study, the radial basis function was used as the kernel function, and the constant of the regularization term in the Lagrangian formula was equal to 1. SVR algorithms were implemented by sklearn package of Python software.

#### 2.4.3 Validation algorithms

To validate the accuracy of inversion results, a LOOCV method and a cross-validation method were used. The basic idea of the LOOCV is to assume that there are N samples, from which N−1 samples are selected for training, and the remaining samples are used for validation, and so on, until all samples are traversed, and the final result is the mean value of N validation errors. It is able to exclude the influence of random factors and ensures that the validation process is repeatable and has the advantage of almost unbiased generalization error estimates (Liu et al., 2011). When using the cross-validation method, RF and SVR build the model by randomly selecting 70% of the samples for model training and the remaining 30% for validation, and the procedure was performed 10 times and the average values were presented here.

Pearson’s coefficient [*R*²; Equation (2)] of determination, root mean square error [RMSE; Equation (3)], relative RMSE [rRMSE; Equation (4)], and mean absolute error [MAE; Equation (5)] were selected as indicators to predict the accuracy of the model.


(2) $$R^{2}=\frac{1-\sum \left(y_{i}-\hat{y_{i}}\right)}{\sum {\left(y_{i}-\bar{y}\right)}^{2}}$$

(3) $$RMSE=\sqrt{\frac{1}{m}}\sum_{i=1}^{m}{\left(y_{i}-\hat{y_{i}}\right)}^{2}$$

(4) $$rRMSE=\frac{RMSE}{\bar{y}}\times 100\%$$

(5) $$MAE=\frac{1}{m}\sum_{i=1}^{m}\left|\left(y_{i}-\hat{y_{i}}\right)\right|$$

where $\hat{y}$ is the predicted value of the model, $y_{i}$ is the sample plot measurement, $\bar{y}$ is the mean value of the sample plot measurement, and m is the number of training and validation sample plots.

## 3 Results

### 3.1 Optimized LiDAR-derived features

**Figure 5:**
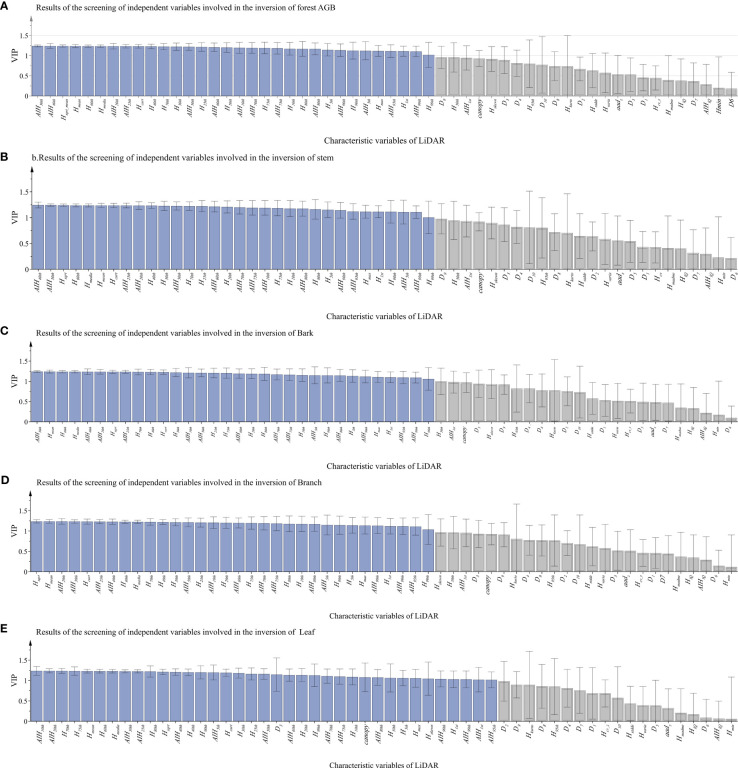
The VIP values of each LiDAR-derived feature graph (the blue ones are selected features with VIP values greater than 1, and the gray ones are abandoned features). From **(A–E)**, the LiDAR characteristics of total biomass, stem, bark, branch and leaf were selected.

To find the best variables to predict the total and component AGB of the forest, observations with VIP value greater than 1.0 were selected for further AGB inversion (Ju et al., 2022). Since PLSR combines the advantages of PCA and MLR, it is effective to extract the reduced by more useful LiDAR-derived features for forest AGBs estimation, especially since input features were not independent of each other. Like the characteristic of PCA, 56 observations and output forest total or each AGB component were projected into new orthogonal spaces, which have better predictive capabilities, and, then, VIP values are calculated and sorted according to the interpreted variance (**Figure 5**). As shown in **Figure 5**, the blue ones are selected features with VIP values greater than 1, and the gray ones are abandoned features. The 37 LiDAR-derived features were selected as features for leaf AGB estimation, and 32 features were selected for total AGB and biomass of other component estimation. Four cumulative height percentiles (*AIH*_30_, *AIH*_40_, *AIH*_20_, and *AIH*_25_) were selected as the top 10 variables for total AGB estimation, and two height percentiles (*H*_8_ and *H*_6_) and four height-related variables (*H*_mean_, *H*_sqrt_, *H*_mad_, and *H*_curt_) were height-related variables.

### 3.2 Forest AGB inversion using RF and SVR

#### 3.2.1 Forest AGB estimation results using RF

**Figure 6:**
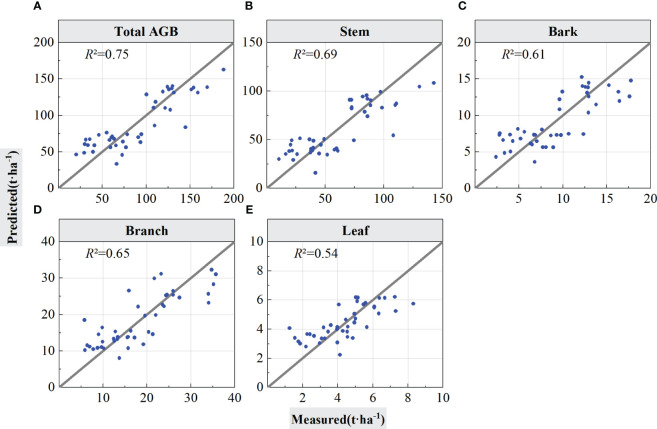
Forest total and component AGB estimation results using LOOCV validation. The solid lines in the scatter plots are 1:1 verification lines: **(A)** for total AGB, **(B)** for stem; **(C)** for bark, **(D)** for branch, and **(E)** for leaf.

The LiDAR features selected by PLSR were input in the RF regression model for forest total biomass and biomass component inversion, and the inversion accuracy of the models was tested using LOOCV and cross-validation. **Figure 6** graphs the estimation results validated using LOOCV, and **Table 4** summarizes the statistical information of the results from **Figure 6**, which are the scatter points for the built models and validations of the models.

**Table 4:** The LOOCV was used to check the accuracy results of the model (RF).

Components	*R* ^2^	RMSE/(t•ha^−1^)	rRMSE/(%)	MAE/(t•ha^−1^)
Stem biomass	0.69	18.14	30.26	14.49
Bark biomass	0.63	2.58	28.34	2.21
Branch biomass	0.65	4.99	27.33	0.65
Leaf biomass	0.54	1.10	25.01	0.87
Total AGB	0.75	22.93	25.30	19.26

As shown in **Table 4**, when using the LOOCV validation method to estimated accuracy of the models, the RF model performed best for total AGB estimation (*R* ^2^ = 0.75, RMSE = 22.93 Mg/ha, rRMSE = 25.30%, and MAE = 19.26 Mg/ha). From the scatter plot of the model fit in **Figure 6**, the prediction results were positively and linearly correlated around the 1:1 line, and it shows some overestimation phenomena for low AGB values, but, with the increase of AGB values, the prediction trend become better with no saturation phenomenon for high AGB values. The accuracy for the stem biomass model (*R* ^2^ = 0.69, RMSE = 18.14 Mg/ha, rRMSE = 30.26%, and MAE = 14.49 Mg/ha) was slightly lower than that of the total AGB. The prediction trend in **Figure 6B** is similar to that in **Figure 6A** but with a slightly lower *R* ^2^. On the basis of the RMSE and MAE values, the error of the estimated component AGB is lower than the error of the total AGB. **Figures 6C–E**, respectively represent the estimation results of forest biomass for stems, branches, and leaves using LOOCV validation.

**Figure 7:**
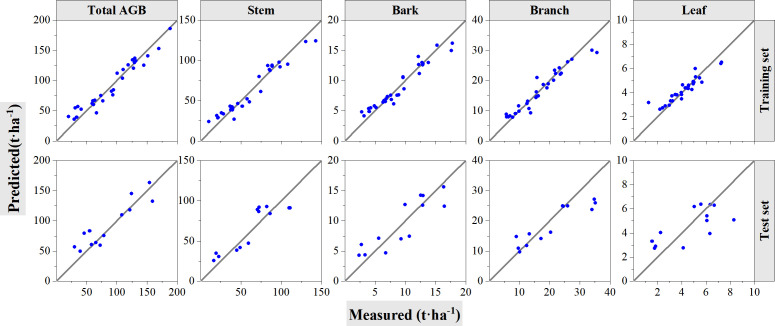
Cross-validation method to test the accuracy of RF models. The solid line is 1:1 verification line. In the figure, the first row shows the performance of the model on the training set. The second row shows the performance of the model on the test set.

**Table 5** and **Figure 7** show the performance of the RF models with training and validation datasets. **Table 5** summarizes the information with model accuracies and validation accuracies. For model training, the constructed RF models showed good estimation results with all *R* ^2^ greater than 0.80 and rRMSEs ranging from 12.66% to 16.21%, whereas the accuracies decreased when the constructed models were validated using the left out 30% samples. *R* ^2^ ranges from 0.49 to 0.65, and rRMSE ranges from 26.37% to 31.89%. According to **Table 5** and **Figure 7**, the RF model for leaf component AGB estimation performed with the lowest *R* ^2^ value during its training procedure does not show the highest rRMSE value although it has the lowest *R* ^2^ value of 0.86. The scatter plots of both the individual components and the total biomass exhibit overestimation and underestimation phenomena. The results may result from the narrow dynamic range of leaf biomass. Meanwhile, the RF model for stem AGB estimation showed better performance even if its highest rRMSE of 16.21% during the model training procedure. However, the model constructed for total AGB estimations acquired highest *R* ^2^ of 0.93 and lowest rRMSE of 12.66% during model training also performed best for validation procedure with highest *R* ^2^ of 0.67 and lowest rRMSE of 25.67%.

**Table 5:** The cross-validation method was used to check the accuracy results of the model (RF).

Components	Training set			Test set		
	*R* ^2^	RMSE/(t•ha^−1^)	rRMSE/(%)	*R* ^2^	RMSE/(t•ha ^1^)	rRMSE/(%)
Stem biomass	0.91	9.92	16.21	0.60	19.15	31.89
Bark biomass	0.90	1.37	14.97	0.57	2.51	29.34
Branch biomass	0.89	2.70	14.84	0.65	4.89	28.32
Leaf biomass	0.86	0.58	13.09	0.49	0.99	26.37
Forest total biomass	0.93	11.89	12.66	0.67	24.56	25.67

The results of both validation methods indicated that the LiDAR characteristic variables were strongly correlated with the total and component AGBs and that the RF model performed good for forest AGBs estimation.

**Figure 8:**
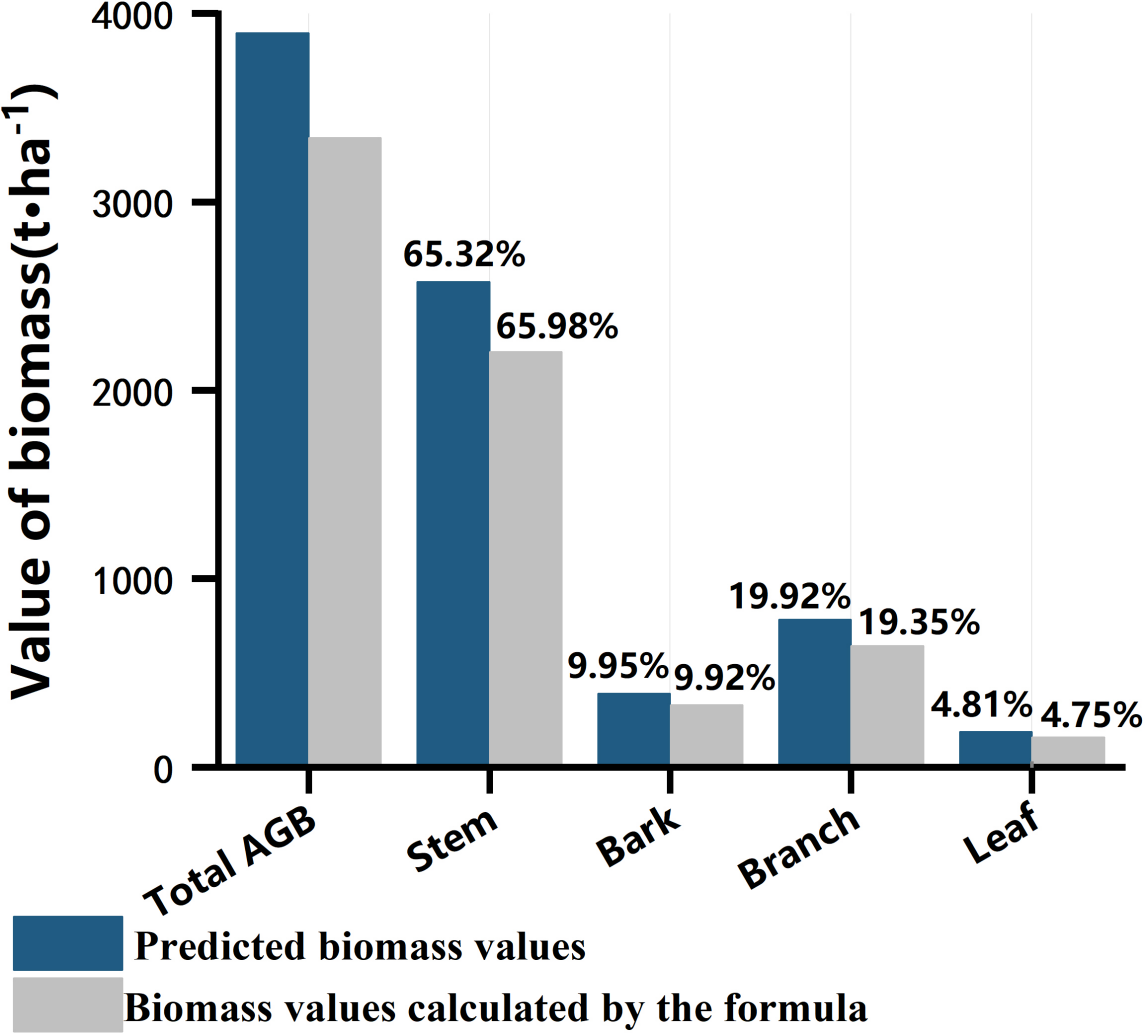
Comparison of total and biomass components of sample plots calculated by the standing wood biomass model with the predicted values from the RF model.

**Figure 8** shows the comparison between the total and biomass components of the sample plots calculated by the stumpage biomass model and the model predictions. The ratios of stem, branch, bark, and leaf in the total biomass from the field collected datasets were 65.98%, 19.35%, 9.92%, and 4.75%, respectively. For the estimated total and component AGBs in the test site, leaf AGB accounts for the lowest ratio with 4.81% and branch and stem accounts for 85.24% of the total AGB. The distribution of each component to the total AGB from the predicted results have similar distribution pattern with the true values.

#### 3.2.2 Forest AGB estimation results using SVR

**Figure 9:**
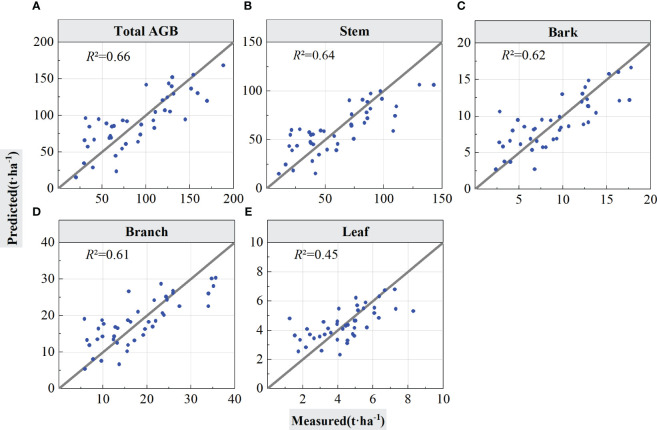
Leave-one-out validation method to test the accuracy of the support vector regression model. The solid line is 1:1 verification line: **(A)** for total AGB, **(B)** for stem, **(C)** for bark, **(D)** for branch, and **(E)** for leaf.

The LOOCV and cross-validation methods were also applied for the validation of estimated AGBs by SVR (**Table 6**, **Figure 9**). Compared with the performance of RF, the overall prediction accuracies of SVR were lower. As shown in **Table 6**, the SVR model performed best for total AGB estimation (*R* ^2^ = 0.66, RMSE = 26.75 Mg/ha, rRMSE = 28.62%, and MAE = 22.00 Mg/ha). From the scatter plot of the model fit in **Figure 9**, the prediction results were positively and linearly correlated around the 1:1 line. Underestimation occurs when AGB values are greater than 150 Mg/ha. Similar as the performance of RF, the *R* ^2^ value for the stem biomass model was slightly lower than that of the total AGB. The scatter plot for stem shows similar trend with total AGB. The lowest *R* ^2^ value was acquired for leaf AGB estimation. The rRMSE values for component AGB estimation range from 27.36% to 32.48%. For total and component forest total biomass estimation, the rRMSE values acquired by SAR were higher than that for the RF algorithms.

**Table 6:** The leave-one-out verification statistics for the support vector regression model.

Components	*R* ^2^	RMSE/(t•ha^−1^)	rRMSE/(%)	MAE/(Mg/ha)
Stem biomass	0.64	19.52	32.48	16.02
Bark biomass	0.62	2.61	28.73	2.07
Branch biomass	0.61	5.30	28.99	4.34
Leaf biomass	0.45	1.20	27.36	0.94
Forest total biomass	0.66	26.75	28.62	22.00

**Figure 10:**
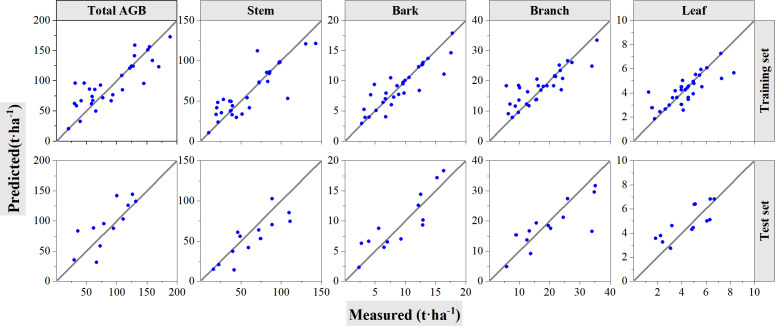
Cross-validation method to test the accuracy of support vector regression models. The solid line is 1:1 verification line. In the figure, the first row shows the performance of the model on the training set. The second row shows the performance of the model on the test set.

For the experiments of cross-validation, 70% of the samples are randomly selected for model training and left 30% are used for validation; the procedures were performed 10 times, and the averaged values were calculated and summarized in **Table 7**. The best performance with the highest *R* ^2^ value was selected among these ten instances and graphed in **Figure 10**. According to the results from cross-validation, the performance of SVR algorithms shows worse performance than RF algorithms. For the model training, *R* ^2^ values range from 0.70 to 0.74, and rRMSE values range from 20.82% to 27.29%. The *R* ^2^ value decreased, whereas the RMSE and rRMSE values increased during testing, with *R* ^2^ ranging from 0.50 to 0.65 and rRMSE ranging from 22.29% to 33.08%. When predicting biomass with the SVR model, the accuracy of validation with LOOCV was 0%–5% higher than that of validation with the cross-validation method. The scatter plots of total and component AGB estimations show no obvious saturation phenomena. The results in **Table 7** revealed that the lower rRMSE values in the model training procedure, the lower rRMSE values in the test procedure.

**Table 7:** The cross-validation method was used to check the accuracy results of the model.

Components	Training set			Test set		
	*R* ^2^	RMSE/(t•ha^−1^)	rRMSE/(%)	*R* ^2^	RMSE/(t•ha^−1^)	rRMSE/(%)
Stem biomass	0.74	16.95	27.29	0.54	19.26	33.08
Bark biomass	0.73	1.85	23.40	0.65	2.48	28.38
Branch biomass	0.71	4.37	24.21	0.57	5.87	31.77
Leaf biomass	0.70	0.91	20.82	0.50	0.87	22.29
Forest AGB	0.76	22.06	24.04	0.56	30.88	31.41

**Figure 11:**
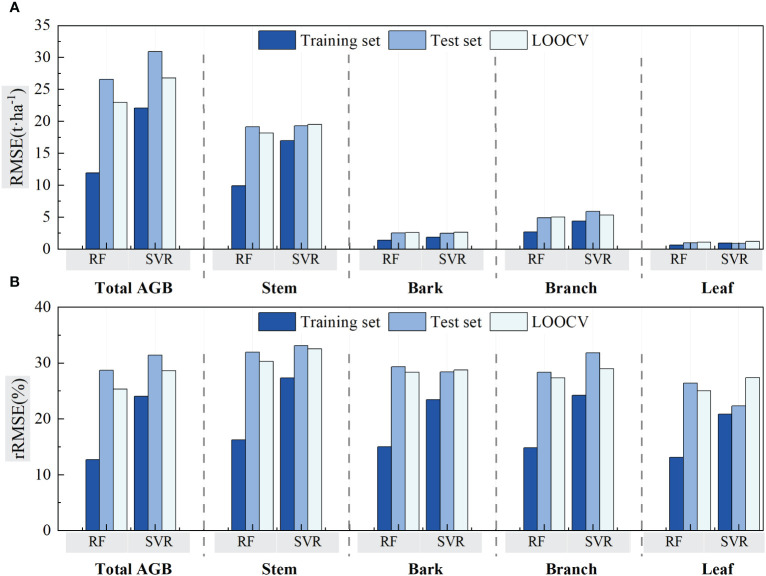
Comparison of the RF and SVR estimated results using LOOCV and cross-validation method. **(A)** The validation method for evaluating two models with RMSE. **(B)** The validation method for evaluating two models with rRMSE.

**Figure 11** compares the RMSE and rRMSE values between RF and SVR algorithms using validation of LOOCV and cross-validation. For both RF and SVR, the RMSE values of the model training are lower than that of LOOCV and model testing in total and component AGB estimations. The RMSE values acquired by the LOOCV method for bark and leaf, which has lower AGB levels, showed a bit higher than that acquired at testing procedures of the cross-validation methods. For stem AGB estimation, RMSE values are almost same for LOOCV and cross-validation test procedure using both RF and SVR algorithms. The RMSE values acquired by LOOCV were lower than that acquired at testing procedure. It seemed that the rRMSE values acquired using the LOOCV method for both RF and SVR algorithms were lower or almost same as that acquired during testing procedures but greater than that acquired at model training procedures. Meanwhile, the rRMSE values for forest total biomass and biomass component estimation ranged from 25% to 32.5%. The acquired values using RF were lower than that using SVR algorithms.

### 3.3 Forest AGB mapping

**Figure 12:**
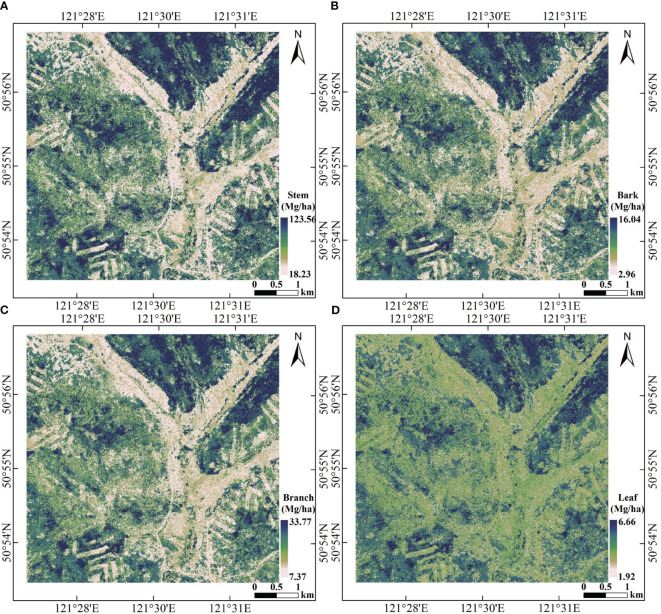
Spatial distribution of biomass component. **(A)** Stem, **(B)** bark, **(C)** branch, and **(D)** leaf.

By comparing the accuracy of the two machine learning models using two validation methods, the RF model was chosen to perform forest total and component AGB inversion for the LiDAR data covering the study area. The spatial distribution maps of component AGBs are shown in **Figure 12**. The stem AGB (**Figure 12A**) ranged from 18.23 Mg/ha to 123.56 Mg/ha, bark AGB (**Figure 12B**) ranged from 2.96 Mg/ha to 16.04 Mg/ha, branch AGB (**Figure 12C**) ranged from 7.37 Mg/ha to 33.77 Mg/ha, and leaf biomass (**Figure 12D**) ranged from 1.92 Mg/ha to 6.66 Mg/ha.

**Figure 13:**
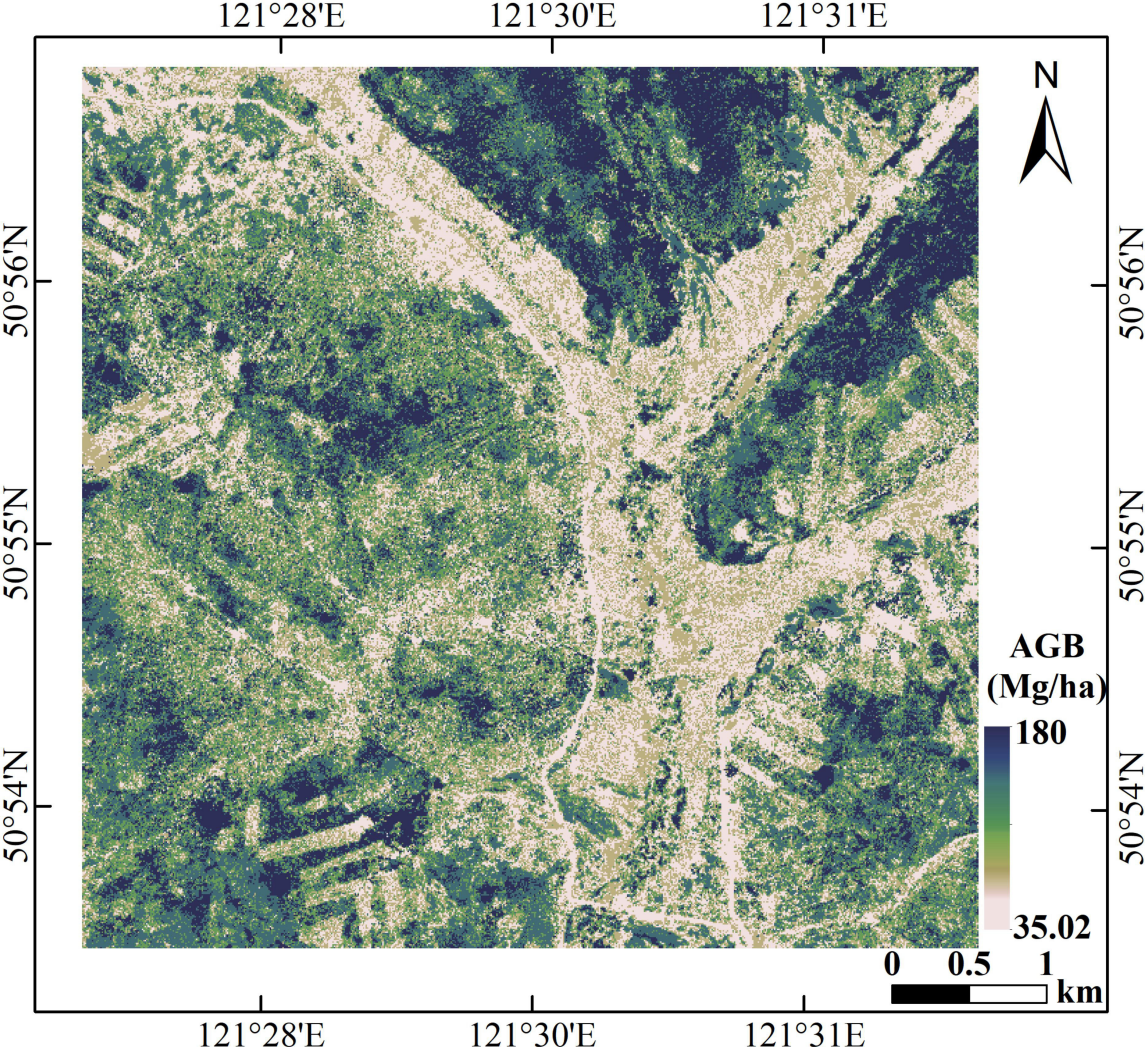
Spatial distribution of forest total biomass in the study area.

**Figure 13** displayed the spatial distribution map of estimated total AGB, which ranged from 35.02 Mg/ha to 180.11 Mg/ha. From **Figures 9** and **10**, the retrieved AGBs seemed consistent with distribution trend of the LiDAR-derived AGB map.

## 4 Discussion

The feature parameters of machine learning models are not limited by dimensionality, and the inversion of forest total biomass using machine learning models has good robustness when there is multicollinearity between feature variables, effectively avoiding the loss of important parameters while maintaining excellent estimation performance (Kotsiantis et al., 2006; Görgens et al., 2015). Therefore, machine learning has been widely used for forest AGB estimation with good accuracy (Cao et al., 2018; Jiang et al., 2022; Li et al., 2022; Torre-Tojal et al., 2022). In the study by Ji et al. (Ji et al., 2022), the accuracy of parametric and non-parametric models for estimating the total forest AGB was compared using SAR data, and the results showed that the non-parametric model gave better estimates than the parametric model, and the non-parametric model was more advantageous than the parametric model in the estimation of the total forest AGB. In our study, two non-parametric models, namely, RF and SVR, were constructed using sample AGB and associated LiDAR remote sensing features for forest total AGB and component AGB inversion. The estimation accuracy of the inverse model was tested by the LOOCV and cross-validation methods.

The results of the comparison between the two machine learning models showed that the *R* ^2^ value (0.75) of the total biomass estimated by RF was higher than that of SVR when using the LOOCV. This result is consistent with the findings of several studies. For example, the RF and SVR models were compared by Görgens et al. and Kumari and Kumar (Görgens et al., 2015; Kumari and Kumar, 2023), and the result was that the RF model outperformed the SVR model in terms of prediction performance. In the work of Görgens et al. (Görgens et al., 2015), neural network, RF, and SVR models were used to predict stand volume in fast-growing plantation forests, and the RF model produced the best prediction results. The study conducted by Kumari and Kumar (Kumari and Kumar, 2023) compared the predictive potential of SVR and RF algorithms in predicting forest AGB. The result was that the predictive performance of RF is better than that of SVR in this study. In these studies above, the RF algorithm achieved better prediction results compared with the SVR. This may be due to the fact that RF is better at handling non-linear relationships and that the model requires fewer hyperparameters to be adjusted compared with SVR. On the other hand, SVR requires selecting appropriate kernel functions and tuning hyperparameters based on the characteristics of the dataset. However, this does not imply that RF outperforms SVR in all scenarios. Therefore, the selection of machine learning methods for inversion of forest AGB needs to be evaluated and compared on the basis of a combination of various factors. In addition, the results of our study using RF to estimate component biomass showed that leaf biomass was the least correlated with LiDAR data, with an *R* ^2^ value of 0.54. This result is in agreement with He et al. (He et al., 2013) who used LiDAR data to estimate the summed component AGB of coniferous forests. In their study, a linear regression model was used, and the results also showed weaker performance for leaf AGB estimation but better performance for stem, branch, and total forest biomass estimation. The range of biomass values of leaves is too low, which may be a reason for the weaker relationship.

Height variables extracted from LiDAR data were strongly correlated with the total and component forest AGBs. In this study, height variables change according to the different component of forest AGBs. *H*_30_, *H*_40_, and *H*_sqt_ are the optimized height variables for total AGB estimation, and the same variables are selected as optimal features for stem estimation as well. *H*_30_, *H*_mean_, and *H*_8_ are the selected optimal height variables for bark AGB estimation, whereas, for branches and leaves, they are *H*_sqt_, *H*_mean_, and *H*_20_ and *H*_10_, *H*_20_, and *H*_9_, respectively. The differences in height variables used for biomass estimation of different components indicate that LiDAR-derived height features have varying explanatory capabilities for biomass composition. It may be related to the extracted height characteristics of LiDAR data to the vertical structure of the forest. In addition, multiple correlations among LiDAR characteristic variables and some overlap among cumulative height percentile variables may also be responsible for the large differences in the relative importance ranking of variables in the component biomass models (Hong et al., 2019). Cao et al. (Cao et al., 2014) estimated total and component biomass in a subtropical forest using small discrete and full waveform airborne LiDAR data. Although the inversion methods that they used were MLR models, their results also confirmed that the height variables extracted from LiDAR data were highly correlated with total forest biomass and component biomass. Forest height features extracted from small-footprint data (Popescu, 2007), small-footprint full-wave form data (Hermosilla et al., 2014a; Hermosilla et al., 2014b), and large-footprint SLICER data (Drake et al., 2002; Lefsky et al., 2005) also explained most of the variability of them for forest structure characteristics.

The comparisons of different validation method are not addressed in other studies, whereas several studies demonstrated that LOOCV performed better especially the limitations of small samples are existent (Zeng et al., 2022; Shi et al., 2023). In this study, the validation difference between LOOCV and cross-validation was compared, and the results revealed that LOOCV showed better accuracy for forest stem, bark, stem, branch, and total AGB estimation but worse accuracy for forest leaf AGB estimation. The accuracy here is related to the value of rRMSE.

Compared with the results of studies using different data sources in the same study area, the height features extracted from LiDAR data outperformed than other data sources. Li et al. (Li et al., 2020) extracted remote sensing features from Landsat8 OLI, Gaofen-1 optical data, and ALOS-1 PALSAR-1SAR to compute forest total biomass at the same test site. They used a fast iterative procedure to optimize input remote sensing features to improve the inversion capability of K-nearest neighbor (KNN) algorithms; the results were validated by LOOCV method; and *R*² = 0.63 and RMSE = 28.84 Mg/ha were weaker than the *R* ^2^ and RMSE values for estimated total AGB using RF and SVR and LOOCV validation in this study. Zeng et al. estimated total and component forest AGBs using features extracted from synthetic aperture radar and demonstrated that C-band polarimetric features performed best for forest leaf AGB estimation with *R* ^2^ = 0.637 and RMSE = 1.27 Mg/ha (Zeng et al., 2022). The estimation of forest total and component AGBs using different data sources revealed the great potential of LiDAR features for accurate estimation.

Although we explored the forest total biomass and biomass component inversion based on optimal LiDAR-derived feature selection with PLSR algorithm and RF and SVR inversion algorithms, there are other machine learning methods that we do not explored in this study. Moreover, the component biomass in this study was calculated by the conversion factors; it may introduce uncertainties for the inversion results; later, the field collected component biomass could be applied in similar study to reduce the uncertainties.

## 5 Conclusions

In this study, 56 height-related features extracted from LiDAR were used in RF and SVR algorithms for forest total and component AGB estimation. PLSR algorithm was utilized for ranking and selecting optimal LiDAR-derived features. LOOCV and cross-validation methods were performed to validate the inversion results obtained by RF and SVR. Four cumulative height percentiles (*AIH*_30_, *AIH*_40_, *AIH*_20_, and *AIH*_25_), two height percentiles (*H*_8_ and *H*_6_), and four height-related variables (*H*_mean_, *H*_sqrt_, *H*_mad_, and *H*_curt_) are more sensitive LiDAR-derived features for total and component forest height estimation. RF performed better than SVR for both forest total biomass and biomass component estimation. LOOCV showed better accuracy for forest stem, bark, stem, branch, and total AGB estimation but worse accuracy for forest leaf AGB estimation. Note that the difference between the validation using LOOCV and cross-validation is no more than 5%. The features extracted from LiDAR showed a weak performance for leaf AGB estimation when compared with other component AGBs and total AGB estimation. Because only 56 height-related features and only two machine learning methods were applied in this study, future work should focus on exploring more metrices especially derived from full-waveform LiDAR data and more machine learning methods like KNN and Gaussian processes.

## Data availability statement

The raw data supporting the conclusions of this article will be made available by the authors, without undue reservation.

## Author contributions

JM: Methodology, Software, Supervision, Validation, Visualization, Writing – original draft. WZ: Conceptualization, Project administration, Resources, Writing – original draft, Writing – review & editing. YJ: Writing – original draft, Writing – review & editing, Validation, Visualization. JH: Validation, Methodology, Formal Analysis, Writing – original draft. GH: Software, Writing – original draft. LW: Visualization, Writing – original draft.
