# Supplementary material for: Total and component forest aboveground biomass inversion via LiDAR-derived features and machine learning algorithms
Source: Front Plant Sci. 2023 Oct 26;14:1258521. doi: 10.3389/fpls.2023.1258521 (PMC10639141; doi:10.3389/fpls.2023.1258521)
Supplement: Supplementary file 1 [file DataSheet_1.docx]

Supplementary Material

# Supplementary Figures and Tables

## Supplementary Figures

|   **Supplementary Figure 1**. Percentage of tree species cover in sample plots. |
| --- |

## Supplementary Tables

**Supplementary Table 1**. LiDAR parameters extracted from airborne LiDAR data.

| **Metrics** | **Parameters** | **Description** |
| --- | --- | --- |
| Variables related to height | Percentile height (*H*_1st_, *H*_5_, *H*_10_, *H*_20_, *H*_25_, *H*_30_, *H*_40_, *H*_50_, *H*_60_, *H*_70_, *H*_75_, *H*_80_, *H*_90_, *H*_95_, *H*_99_) | The normalized point cloud data is sorted by percentile height. |
|  | Cumulative height percentile *(AIH*_1_, *AIH*_5_, *AIH*_10_, *AIH*_20_, *AIH*_25_, *AIH*_30_, *AIH*_40_, *AIH*_50_, *AIH*_60_, *AIH*_70_, *AIH*_75_, *AIH*_80_, *AIH*_90_, *AIH*_95_, *AIH*_99_) | Sort all normalized point clouds in the statistical unit by height and calculate their cumulative height. |
|  | Average absolute deviation (*aad_*_z_) | $\frac{\sum_{i=1}^{n} \left\vert Z_{i}-\bar{Z} \right\vert}{n}$ |
|  | Canopy undulation rate (*H*_crr_) | $\frac{mean-min}{max-min}$ |
|  | Cumulative height percentile interquartile spacing（*AIH*_IQ_） | $AIH75\%-AIH25\%$ |
|  | Coefficient of variation (*H*_cv_z_) | The coefficient of variation of heights of all first returns (Liu et al., 2018) |
|  | Kurtosis of heights (*H*_kurt_) | Median height above ground of all first returns (Zhou et al., 2022) |
|  | Median absolute deviation of media (*H*_media_) | The maximum value of the Z value of all points in the statistical cell. |
|  | Maximum height (*H*_max_) | The Minimum value of the Z value of all points in the statistical cell. |
|  | Minimum height (*H*_min_) | In the statistical cell Average of Z values of all points. |
|  | Average height (*H*_mean_) | Median z-value of all points in the statistical cell. |
|  | Median of the average (*H*_mad_) | $\sqrt[3]{\frac{\sum_{i=1}^{n} {Z_{i}}^{2}}{n}}$ |
|  | Power of three average (*H*_curt_) | $\sqrt[2]{\frac{\sum_{i=1}^{n} {Z_{i}}^{2}}{n}}$ |
|  | power of two average (*H*_sqrt_) | $Elev75\%-Elev25\%$ |
|  | Height percentile interquartile spacing (*H*_IQ_) | Symmetry of the distribution of Z values of all points within a certain statistical cell. |
|  | Height skewness (*H*_skewn_) | Standard deviation of normalized height of all points above stated in meters threshold (Michałowska and Rapiński, 2021) |
|  | Standard deviation (*H*_stddev_) | The variance of the heights of all first returns (Michałowska and Rapiński, 2021) |
|  | Variance of height (*H*_variance_) | The kurtosis of the heights of all points (Liu et al., 2018) |
| Density-related variables | Density variables (*D*_10_, *D*_20_, *D*_30_, *D*_40_, *D*_50_, *D*_60_, *D*_70_, *D*_80_, *D*_90_, *D*_100_) | The proportion of points above the quantiles to total number of points (Zhou et al., 2022) |

Note: All the above data are the data of the first returns of LiDAR. $Z_{i}$ is the height value of the ith point in each statistical cell, $\bar{Z}$ is the average height of all points in each statistical cell, $n$ is the total number of points in each statistical cell, $mean$ is the average height of all points in each statistical cell, $min$ is the minimum height value of all points in each statistical cell, $max$ is the maximum height value of all points in each statistical cell, $AIH75\%$ is the 75% cumulative height percentile, $AIH25\%$ is the 25% cumulative height percentile, $Z_{std}$ is the standard deviation of the height values of all points in each statistical cell, $Z_{mean}$ is the average height of all points in each statistical cell, $Elev75\%$ is the 75th percentile of height, is the 25% height percentile.
